# Genomic Organization and Control of the Grb7 Gene Family

**DOI:** 10.2174/138920208783884847

**Published:** 2008-03

**Authors:** E Lucas-Fernández, I García-Palmero, A Villalobo

**Affiliations:** Instituto de Investigaciones Biomédicas, Consejo Superior de Investigaciones Científicas & Universidad Autónoma de Madrid. Arturo Duperier 4, E-28029 Madrid, Spain

**Keywords:** Adaptor proteins, cancer, congenital disorders, diabetes, genetic imprinting, Grb7, Grb10, Grb14, uniparental disomy, splice variants.

## Abstract

Grb7 and their related family members Grb10 and Grb14 are adaptor proteins, which participate in the functionality of multiple signal transduction pathways under the control of a variety of activated tyrosine kinase receptors and other tyrosine-phosphorylated proteins. They are involved in the modulation of important cellular and organismal functions such as cell migration, cell proliferation, apoptosis, gene expression, protein degradation, protein phosphorylation, angiogenesis, embryonic development and metabolic control. In this short review we shall describe the organization of the genes encoding the Grb7 protein family, their transcriptional products and the regulatory mechanisms implicated in the control of their expression. Finally, the alterations found in these genes and the mechanisms affecting their expression under pathological conditions such as cancer, diabetes and some congenital disorders will be highlighted.

## INTRODUCTION

The so-called adaptors form an ample group of proteins that generally lack enzymatic activity, but play an important role in multiple signal transduction processes in the cell. They function by bringing together in a proper conformational manner, different components of given signaling pathways to achieve their correct functionality in transmitting signals along defined routes. Among the adaptors found in mammalian cells, the growth factor receptor bound protein 7 (Grb7) family that is formed by three members: Grb7, Grb10 and Grb14, has acquired certain relevance. These proteins mediate the interaction between many activated (phosphorylated) tyrosine kinase receptors located at the cell surface, from this its acronym Grb pertaining to Growth factor receptor bound, and their downstream signaling effector proteins came into existence. In addition, these adaptor proteins are able to interact with other non-receptor tyrosine-phosphorylated proteins involved in signaling events (see [1-4] for reviews and references therein).

The mammalian Grb7 family members show significant homology to the *Caenorhabditis elegans* protein denoted Mig10, which is implicated in embryonic neuronal cell migration [5]. These proteins exhibit a conserved multidomain structure. Hence, in their central section they present a region termed GM, for Grb and Mig, that includes a Ras-associating (RA) domain, which allows for their interaction with members of the Ras superfamily of small G proteins; and a pleckstrin homology (PH) domain whose function is to allow their binding to cell membrane phosphoinositides, and where our group has additionally identified (in Grb7) a functional calmodulin-binding domain overlapping its proximal region [6]. In their N-terminus, these proteins present a proline-rich (PR) domain for interaction with proteins containing a Src homology 3 (SH3) domain. Distal to the GM region is located the BPS domain (for between PH and SH2), which is responsible for interaction with the insulin and insulin-like growth factor-I (IGF-I) receptors and finally, the C-terminus contains the all-important Src homology 2 (SH2) domain, whereby the Grb7 family proteins interact with phosphotyrosine residues present in activated receptors and signaling proteins. The *C. elegans* Mig10 protein lacks, however, an SH2 domain and contains instead an additional PR domain in its C-terminus (see [1-4] for reviews and references therein).

Grb7 interacts with many tyrosine kinase receptors, including the epidermal growth factor receptor (EGFR/ErbB1) and others, erytoblastic leukemia viral oncogene homolog receptors (ErbB2, ErbB3, ErbB4) just to give some examples of a set of receptors involved in tumor biology and it has been implicated in cellular migration because of its interaction with focal adhesion kinase (FAK) and the erythropoietin-producing hepatocellular carcinoma cells receptor B1 (EphB1), an ephrin receptor. This adaptor protein also plays a prominent role in the metastatic spread of tumor cells, where it is frequently overexpressed (see [1-4, 7] for reviews and references therein). In addition, Grb7 appears to be involved in cellular processes controlling angiogenesis [6], and its role in the negative control of protein translation has recently been shown [8].

Grb10 translocates to the plasma membrane upon insulin stimulation and binds with high affinity to the insulin receptor, and also with the IGF-I receptor, thereby inhibiting their tyrosine kinase activity. Grb10 also acts as a positive regulator of phosphatidylinositol 3-kinase (PI3K)/Akt (for v-*akt* murine thymoma viral oncogene homolog)-mediated cellular functions and it has been shown to associate to the mitochondrial outer membrane in response to IGF-I or serum treatment, from where it could exert an anti-apoptotic action. Grb10 possibly regulates the ubiquitination of target proteins, such as tyrosine kinase receptors, because of its interaction with Nedd4, a ubiquitin protein ligase. Furthermore, Grb10 seems to play a leading role in development by negatively affecting cell proliferation because of its actions on the signaling pathways, which are the insulin and IGF-I receptors, but by the growth hormone receptor as well (see [1-4, 9-11] for reviews and references therein). Nevertheless, the negative control of Grb10 on mitogenesis is not a universal event, since a stimulatory role of this protein on cell proliferation mediated by platelet-derived growth factor-BB (PDGF-BB), insulin and IGF-I has also been reported [12].

Grb14 exerts its functional roles upon its association with the insulin and the fibroblast growth factor receptors, thereby inhibiting both cell proliferation and insulin-mediated glycogen synthesis (see [1-4, 13] for reviews and references therein).

As we have seen, the Grb7 protein family members control numerous cellular functions because of the variegated spectrum of receptors they interact with and the multiplicity of receptor-controlled signaling pathways in which these adaptor proteins intervene. In order to illustrate the global physiological impact of these proteins on the control of cellular functions, Fig. (**1**) summarizes the main biological processes in which the Grb7/Grb10/Grb14 proteins appear to play a relevant role.

## THE *GRB7* GENE

Human *GRB7* maps to the large arm of chromosome 17 at the 17q12-q21.1 locus (see Fig. (**2**) left panel), near to the *ERBB2* gene within the 17q12 amplicon, which is also called the *ERBB2 *amplicon [14, 15]. Transgenic mouse models expressing activated ErbB2 under the control of its endogenous promoter(s), but not under the control of a mouse mammary tumor virus-based promoter, express high levels of Grb7 and other genes within the same *ERBB2* amplicon, such as *CAB1* [16]. The major human *GRB7* mRNA transcript is 2.4 kb long and is accompanied by a less abundant transcript of 4.1 kb [15, 17]. The *GRB7* gene comprises 15 exons, of which only 14 have total or partial coding capacity, yielding three transcripts of 2,107, 2,197 and 2,228 pb that differ only in their 5’-untraslated region (5’-UTR) and all encoding the same 532 amino acids (59.7 kDa) Grb7 protein. The C-terminal truncated variant Grb7V lacking the SH2 domain has 447 amino acids (49.4 kDa) and is encoded by a 1,667 pb transcript expressed by an altered gene with only 13 exons of which only 12 have total or partial coding capacity [18, 19].

Mouse *Grb7* is located in chromosome 11 [14, 20] but in rats this gene maps in chromosome 10 [21, 19]. In pig, *GRB7* maps in chromosome 12 [22] and in chimpanzee is located in chromosome 17 like in human [19, 21]. The human and mouse chromosomal loci where *GRB7* reside, are evolutionarily conserved regarding the order and orientation of the genes they contain [20]. Murine *Grb7* mRNA is highly expressed in liver and kidney and to a lesser extent, in testis and ovary with trace levels in lung, yielding a transcript of approximately 2.3 kb [23].

Among the new uncovered functional roles of the Grb7 protein, it has been recently described that it can act as a translational repressor upon binding to the 5’-UTR of a targeted mRNA, thereby blocking the recruitment of the translation eukaryotic initiation factor 4E (eIF4E) and silencing protein expression [8].

## IMPLICATION OF *GRB7* IN CANCER

The *GRB7* and *ERBB2* genes are co-amplified and/or overexpressed in human gastric and esophageal carcinoma cell lines, and in primary gastric and esophageal cancers, particularly within the lower segment of the esophagus [14, 15, 24-26]. In some human esophageal carcinoma patients, amplification of the *GRB7* gene was not detected but its mRNA was overexpressed and co-expressed with the *ERBB2* and/or *EGFR* genes [27, 28]. In contrast, a positive correlation between an increased number of copies of the *GRB7* gene and elevated mRNA expression levels was found in gastric cancers [29]. The aforementioned Grb7 truncated isoform Grb7V, in which the SH2 domain was substituted by a short hydrophobic tail, was detected in highly invasive human esophageal carcinomas, presenting a particularly enhanced expression in metastasized lymph nodes, underscoring the role of the Grb7 protein in tumor invasion [18]. Genomic hybridization analysis using cDNA microarrays detected the co-amplification of the *ERBB2* and *GRB7* genes in a series of human gastric cancer xenographs, primary tumors and tumor cell lines, although overexpression was apparent for *ERBB2* but not for *GRB7 *[25].

Co-amplification of *ERBB2* and *GRB7* was also found in human breast cancer cell lines [17], and has been additionally detected in non-invasive ductal carcinomas *in situ *[16]. Increased expression of *GRB7* correlates with a lower survival rate in breast cancer patients with tumors with either high or low expression of *ERBB2* [30, 31]. A subset of the estrogen receptor (ER)-negative breast cancer subtype presents overexpression of the genes within the *ERBB2* amplicon including *GRB7 *[32].* GRB7* expression not only positively correlates with *ERBB2* expression in human breast carcinoma but with *ERBB3* expression as well [33], although the latter maps at chromosome 12q13 [34]. Nevertheless, no association was found between common single-nucleotide polymorphisms (SNPs) in genes within the *ERBB2 *amplicon and the risk of developing breast cancer in a study conducted in over two thousand patients when compared to a similar number of control subjects [35].

The *GRB7* gene also co-amplifies with additional genes located within the *ERBB2* amplicon such as *CAB1, A39, C51 *and *MLN64* in gastric and breast cancers [17, 24]. However, the minimum region of recurrent amplification within the 17q12 amplicon includes just the *STARD3, ERBB2* and *GRB7* genes in human breast cancer [36].

An increased *GRB7* gene copy number was also detected in human testicular germ cell tumors, exhibiting a particularly high expression in immature teratoma, embryonal carcinoma, choriocarcinoma and some (but not all) seminoma, in contrast to its rare incidence in normal testicular tissues [37, 38]. In fact, *GRB7* is approximately 5-fold downregulated in most seminomas as compared to embryonal carcinomas [39]. However, no elevated *ERBB2* expression was found in these testicular tumors [37]. Instead, there is a positive correlation between the co-expression of *GRB7* and *KIT* (a gene encoding the v-*kit* Hardy-Zuckerman 4 feline sarcoma viral oncogene homolog receptor, another tyrosine kinase receptor that interacts with Grb7 and which maps at the locus 4q11-q12) in seminomas, but not in non-seminoma testicular tumors. In the latter, however, there was a positive correlation between the co-expression of *GRB7* and *KRAS2*, a gene encoding a small G protein of the Ras family, which maps to the 12p12.1-p11.2 locus [40].

The expression of the *GRB7* gene was also increased in cells from chronic lymphocytic leukemia patients, in which the highest expression levels were found in the advanced stages of the disease (stage IV) as compared to less advanced stages (stage I) [41].

## THE *GRB10* GENE

In human, as in chimpanzee, the *GRB10* gene is located in chromosome 7 (see Fig. (**2**) center panel). The DOPA-decarboxylase (*DDC*) and Cordon-bleu (*COBL*) genes flank it and maps at the 7p11.2-p12 locus (or as described by others at the 7p11.1-p12 or 7p11.2-p12.2 locus), close to any event of the *EGFR* gene, which is located at ≈ 3.7 Mb toward the centromere [19, 21, 42-45]. This gene is alternatively spliced yielding transcripts that may encode a protein with a partial deletion of the PH domain and/or an N-terminal sequence addition. High expression of a prominent 4.7 kb transcript for the human *GRB10* splice variant denoted GRB10/IR-SV1 (insulin receptor bind spliced variant 1) (hGrb10α), a 548 amino acid (61.9 kDa) protein, with a deletion of 80 amino acids in the PH domain and a distinct N-terminus addition as compared to the mouse Grb10, are found in skeletal muscle and pancreas, and in lesser quantities in heart, brain, placenta, lung, liver and kidney [46]. A second *GRB10* 5.6 kb transcript encoding a 536 amino acid (60.7 kDa) protein with an intact PH domain named GRB-IRβ/GRB10 (hGrb10β) was later described and its expression also detected to be elevated in pancreas and skeletal muscle although it is also present in most tissues [47]. A third isoform of 594 amino acids (67.1 kDa) denoted hGrb10γ, containing the same N-terminal extension as hGrb10α but with an intact PH domain, was also described [43]. Its expression was also high in skeletal muscle and in some tumor cells, as HeLa cells and diverse breast cancer cell lines [43]. The detailed cloning and sequence analysis of the human *GRB10* gene shows the presence of 17 exons (or 22 if subdivided exons are considered) among translated and untranslated ones (see [19]), of which 14 participate in the translation of all isoforms described up to day, including not only the mentioned hGrb10α, hGrb10β and, hGrb10γ but also the additional described hGrb10ζ, hGrb10ε and hGrb10σ isoforms, while 3 additional exons are translated in at least one of the isoforms [48].

The mouse *Grb10* gene maps to chromosome 11, and is also flanked by the *Ddc* and *Cobl* genes. Like its human ortholog, *Grb10* is in the proximity of the *Egfr* gene, located at a distance of ≈ 4.9 Mb toward the centromere [44, 49]. It was initially proposed that mouse *Grb10* encodes several isoforms of the protein (mGrb10) because of the existence of alternative translational start sites [49]. The most abundant transcripts are 5.5 kb and 1.5 kb long and the transcription of the mGrb10α and mGrb10δ isoforms is initiated at exon 1, although the mGrb10δ isoform originates from a spliced variant transcript lacking exon 5 as compared to the full-length transcript encoding the mGrb10α isoform [50]. The start of the translation occurs in exon 3 and ends at exon 18. The correlation between *Grb10* exons and the protein functional domains is as follows: exon 4 encodes the PR domain, exons 10-13 the PH domain, exons 13-16 encode the BPS domain and part of the SH2 domain and exons 16-18 the rest of the SH2 domain [50].

The expression of two prominent *GRB10* transcripts was also detected in the adipose tissue of the rhesus monkey [46]. Overexpression of *Grb10 *mRNA occurs in fetal rat liver as compared to adult liver, where it nearly disappears, suggesting a role of this gene in tissue growth [51]. This was further supported by the enhanced expression of the *Grb10* gene found in fast growing cultured pre-implantation mouse embryos (blastocytes) [52].

## IMPRINTING OF THE *GRB10* GENE

Genetic imprinting consists in the selective expression of a single allele of a given gene either that residing in the mother or in the father homologous chromosome, what results in a non-Mendelian inheritance pattern. The mechanism of imprinting may underlie the methylation of CpG dinucleotides lying in selected sectors of the gene such as the promoter region, catalyzed by specific DNA methyl-transferases; and/or chromatin modification, involving the posttranslational acetylation and/or methylation of histones bound to the targeted gene catalyzed by histone acetyl-transferases and methyl-transferases, respectively. These processes usually occur at the gametes and the imprinted mark is thereafter transmitted during embryonic development and through the subsequent developmental stages up to adulthood. In general, these differential DNA and histone modifications result in the epigenetic silencing of the targeted genes.

The human *GRB10* gene may be imprinted either paternally or maternally, or expressed from both alleles depending on the tissue and the protein isoform under consideration. Thus, in human fetal brain, the paternal allele is the one transcribed for most of the Grb10 isoforms, while in skeletal muscle, the transcribed allele for the Grb10γ1 isoform is from maternal origin [48], whereas biallelic expression was detected in other fetal peripheral tissues [53]. These and other authors noticed that the paternal-specific expression of human *GRB10* in the fetal central nervous system (brain and spinal cord) appositely differs from the maternal allele-only transcription in the mouse brain [48, 53]. Despite the imprinted nature of *GRB10*, no allele-specific methylation of most of the 5’ CpG island of this gene in human fetal brain was found by these authors [48] and the silenced mouse *Grb10* allele remained inactive despite the exposure of cells to inhibitors of DNA methylation and of histone deacetylases [54]. Additional work revealed the maternal expression of *Grb10* in mouse embryos, adult kidney and liver; while in adult brain the paternal allele was mainly expressed [55]. Also, *Grb10* is paternally imprinted in bovine embryos, being expressed in both *in vitro*-fertilized and parthenogenetically activated blastocysts [56].

Uniparental disomy (UPD) consists in the inheritance of two homologous copies of a given chromosome (or part of a chromosome) from a single parent, either the mother or the father. This is most likely due to the rescue-driven loss of a third copy of the chromosome in an otherwise non-viable trisomic zygote, among other mechanisms. As earlier reviewed, paternal uniparental disomy (patUPD) affecting the mouse *Grb10* gene results in embryonic and postnatal overgrowth while maternal uniparental disomy (matUPD) results in undergrowth [57].

Within the promoter region of mouse *GRB10 *are located two major 1.4 kb long CpG islands, the first encompassing exon 1a and the second encompassing exon 1b [50, 55]. These CpG islands are also present at analogous positions in human *GRB10*, presenting notorious sequence homologies in both species, except for a 600 bp mouse-specific tandem repeat in the promoter region of the parentally expressed gene [55, 58]. Both in mouse and human, DNA methylation at the *GRB10* promoter was not different in the paternal and maternal expressed alleles in the first CpG island, which was hipomethylated, but increased methylation was found in the second CpG island in the maternal allele as compared to the paternal one [55, 58]. Most recently, however, a third CpG island was identified around the subdivided exon 1c in mouse *Grb10* that exhibits extensive methylation in both maternal and parental alleles in neurons and glial cells [59]. Two distinct promoters, a major one active in most tissues and a brain-specific promoter located downstream of the first one were thus proposed to exist in the mouse and human *GRB10* genes [55]. In mouse, methylation of the CpG islands was not only detected during oocyte growth but they kept extensively methylated into adulthood [60]. Most interestingly, differential acetylation and methylation of histones in the nucleosomes containing the CpG islands 1 and 2 in the promoter of the parental alleles of the mouse *Grb10* gene have been detected in neurons, glia and fibroblasts, what may contribute to the epigenetic control of the expression of this gene [59].

As a consequence of imprinting, a deviation of a Mendelian 1:1 female/male transmission ratio of the grandparental alleles in the paternal chromosome containing the imprinted gene *GRB10 *was found in females as compared to males where the fraction of the transmitted grandmaternal alleles was 0.38 and 0.5 respectively due to uneven loss of human embryos in the former [61].

## IMPLICATION OF *GRB10* IN PATHOLOGICAL PROCESSES

Several connections between Grb10 and cancer have been reported. Thus, *Grb10* was shown to be overexpressed more than three-fold in mouse cells infected with tumorigenic adenovirus 12 as compared with cells infected with non-tumorigenic adenovirus 5 [62]. Also, increased expression of *GRB10* mRNA was detected in primary cervical squamous carcinomas in human as compared to normal uterine squamous cell tissues [63]. Furthermore, dysregulation of *GRB10* has been detected in human metastatic malignant melanomas [64].

Several transgenic mouse lines with ectopic overexpression of *Grb10* have been shown to present growth retardation after weaning and hyperinsulinemia due to insulin resistance [65]. Conversely, knockout mice with a disrupted *Grb10* gene exhibited body overgrowth, particularly due to muscle mass increase accompanied by decreased adiposity, as well as increased glucose tolerance, insulin sensitivity and insulin-mediated cellular signaling [66, 67]. These observations appear to link Grb10 to the pathogenesis of type 2 diabetes. In this context, it has been shown that some SNPs in the *GRB10* gene correlates indeed with type 2 diabetes in human [68].

Albeit the important role of Grb10 in organismal development, and in contrast to the RET tyrosine kinase, which is one of its upstream signaling receptors, mutations in *GRB10* were not linked to Hirschsprung disease, a congenital condition characterized by the absence of intrinsic enteric ganglion cells in some sectors of the gastrointestinal track, including the colon, which results in a distended atonic megacolon [69].

The Silver-Russell syndrome (SRS) is an inherited condition characterized by intrauterine and postnatal growth retardation resulting in typical dysmorphic features, such as a small triangular face with down-turned corners of the mouth, prominent forehead, skeletal asymmetry of the trunk and limbs, and clinodactyly of the fifth finger, which consists in its inner bending due to an undeveloped middle phalange [70]. Multiple genetic alterations have been associated with SRS. Among them and of particular importance to us are: the maternal uniparental disomy of chromosome 7 (matUPD7) occurring in 7-10 % of SRS patients, in which disruption of genomic imprinting takes place; and a few patients where maternal duplication of the 7p11.2-p13 region was detected [70-72]. Although *GRB7* maps close to a translocation breakpoint in chromosome 17 detected in three SRS patients, the implication of the *GRB7* gene in this syndrome was soon discarded [73].

The first indication, however, of the possible implication of *GRB10* as a candidate gene for the SRS was obtained in the mouse, where the imprinted gene *Meg1/Grb10* was associated with the maternal or paternal duplication of proximal chromosome 11, where this gene is located, which was correlated with prenatal growth retardation or growth promotion, respectively [74]. Soon thereafter, sub-microscopic duplication of the 7p12.1-p13 region of human chromosome 7, which includes the *GRB10* gene among others, was detected in mothers and daughters with SRS phenotypes [75, 76], although this duplication was found in only 2.4 % of 32 additional SRS patients screened [77]. Interestingly, a partial matUPD7 was described in an SRS patient in which only a small segment of matUPD7 occurred while the rest of chromosome 7 was of biparental inheritance, including *GRB10* [78]. This particular segmental matUPD7 led to suggest that *GRB10* may not be central to SRS etiology [71], although SRS patients with three *GRB10 *copies were later detected [79]. Also, against the implication of *GRB10* in the etiology of SRS it has been argued that the imprinting of this gene in brain and skeletal muscle is incomplete and isoform-specific, and that there is absence of *GRB10 *imprinting in the growth cartilage plates, a tissue involved in very active growth in young subjects [80].

Also, mutations in *GRB10* accounting for a proline to serine substitution at residue 95 of Grb10 were detected in two out of 58 screened SRS patients [81], although an additional study did not reveal *GRB10* mutations in a subset of 18 SRS patients with non-matUPD7 [53]. In contrast, although frequent SNPs were found in exons 3 and 12, and intron 3, as well as a heterozygous microsatellite repeat in intron 14 of the *GRB10* gene of SRS patients, no other alterations have been detected, further suggesting to those authors that this gene does not play a major role in the etiology of SRS [48]. Furthermore, no epimutations in the differentially-methylated region of the *GRB10* gene were found in a large set of 46 screened SRS patients performed in two independent studies [58, 82].

## THE *GRB14* GENE

Using fluorescence *in situ* hybridization, the *GRB14* gene was located in the long arm of human chromosome 2, most precisely in the 2q22-q24 locus (see Fig. (**2**) right panel), relatively close to the *ERBB4* gene located at the 2q33.3-q34 locus [83]. These authors also detected minor hybridization signals at 12q13 and 6q27, which they suggested were derived from other *GRB7* related genes [83]. The *GRB14 *gene comprises 14 exons, which yield the 2,382 bp *GRB14 *transcript that encodes for the 540 amino acid residues (60.9 kDa) protein (see [19, 21]). The human *GRB14* mRNA is highly expressed in liver, kidney, pancreas, ovary, testis, heart and skeletal muscles and it is also very abundantly present in kidney embryonic cells, some prostate cancer cell lines and breast cancer cells [84], particularly in ER-positive cell lines [84, 85]. Originally, three most prominent mRNA transcripts of 2.3, 2.4 and 2.5 kb were detected, which were frequently accompanied by a less abundant 9.5 kb transcript [84]. 

The chimpanzee and mouse *GRB14* orthologs are located in chromosome 2, whereas the rat gene maps in chromosome 3 (see [19]). In both mouse and rat, Grb14 is a 538 amino acid protein highly expressed in insulin-responsive tissues such as liver, heart, skeletal muscle, pancreas and white adipose tissues, as well as in the brain, and is encoded by a major 2.5 kb transcript and a lesser 1.9 kb transcript [86]. Mouse Grb14 bears 93 % sequence identity to rat Grb14 and 85 % sequence identity to human Grb14 [87].

## IMPLICATION OF *GRB14* IN PATHOLOGICAL PROCESSES

The *GRB14 *gene is highly transcribed in human breast and prostate tumor cells [84, 85], which might underscore its potential implication in cancer biology. Moreover, a high mutation frequency of this gene has been found in human colorectal cancers showing microsatellite instability (30 % of primary tumors and 50 % of tumor cell lines), as they are prone to failure in repairing errors occurring during DNA replication [88]. *GRB14* expression is under hormonal control since it is downregulated by estradiol and upregulated by insulin [85], and overexpression of *GRB14* has been detected in animal models and human patients exhibiting insulin resistance due to type 2 diabetes [89].

## FUTURE PERSPECTIVES

The study of the organization and control of the genes encoding the three Grb7 family members is still in its infancy. Among the relevant issues that still require great experimental attention we should mention the full characterization of the promoter regions of *GRB7, GRB10, *and *GRB14*, the complete identification of the panel of transcription factors and accessory proteins involved in their expression and the potential role of iRNAs controlling the translation of their respective transcriptional products. Among the specific issues related to the alterations observed in the organization of these genes, it should be of interest to unravel the underlying mechanisms of amplicon duplication involving the *GRB7* and *ERBB2* genes, as observed in some human cancers (see [1-3, 7] for reviews and references therein); as well as the mechanisms responsible for the observed segmental UPD of chromosome 7 in a number of Silver-Russell syndrome patients that, although appears not to affect the *GRB10* gene [78], might clarify the basis of this congenital defect. Given the negative control exerted by the Grb10 and Grb14 proteins on cell proliferation (see [1-4, 9-11, 13] for reviews and references therein), it should be of interest to perform a systematic screening of tumors in search of disabling mutations affecting the *GRB10* and *GRB14* genes, in order to determine whether they might work as tumor suppressor genes. Alternatively, and given that Grb10 also appears to exert a promitogenic function in some context [12], it should be of interest to explore whether mutations in the *GRB10* gene, in addition to those already known, could be of relevance to explain the high proliferation rate of some tumors. Poly(ADP-ribose) polymerases catalyze the poly-ADP-ribosylation of proteins, including those involved in the control of the maintenance of genomic functionality and integrity, because of their participation in single- and doublestrand DNA repair, recombination and replication, gene transcription and control of telomeric functions [90]. Tankyrase-2, which belongs to this group of poly(ADP-ribose) polymerases, has been shown to bind to the Grb14 protein [91]. As this enzyme has a relevance in telomere maintenance [90], further work should be performed to determine whether or not the *GRB14* gene might control processes related to cellular senescence. Finally, given the variety of signaling pathways in which the three family members Grb7/Grb10/Grb14 appear to be involved, it may not be out of the ordinary that mutations and/or rearrangements affecting their encoding genes, and hence the stability of their transcripts and/or the functionality of their protein products, are found in additional congenital pathological processes in human and/or animal models, and hence clarifying their etiologies. A prediction that only a sustained experimental effort in this direction may only confirm this or contrarily falsify.

## Figures and Tables

**Fig. (1):**
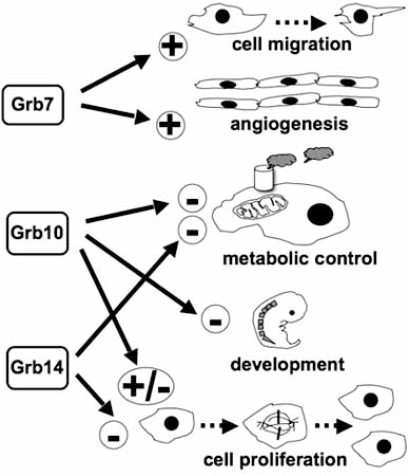
**Control exerted by the Grb7/Grb10/Grb14 proteins on cellular and physiological processes.** The cartoon represents a selected series of cellular and physiologically relevant processes in which the Grb7 family member proteins exert a positive or negative control by participating in defined signaling pathways. These processes are: cell migration (Grb7), angiogenesis (Grb7), metabolic control (Grb10 and Grb14), development (Grb10), and cell proliferation (Grb10 and Grb14). Encircled plus and minus symbols represent, respectively, activation and inhibition of the indicated processes. See text for additional details.

**Fig. (2):**
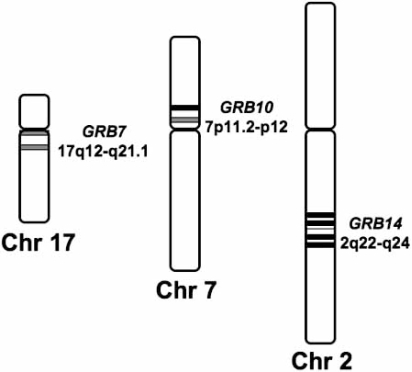
**Chromosomal localization of the human *GRB7*, *GRB10* and *GRB14* genes.** The cartoon represents partial ideograms of human chromosomes (Chr) 17, 7 and 2 where the *GRB7*, *GRB10*, and *GRB14* genes are respectively located. The approximate distribution of chromosome bands corresponding to the loci: 17q12- q21.1 (for *GRB7*), close to the centromere in the long arm of chromosome 17; 7p11.2-p12 (for *GRB10*), close to the centromere of the small arm of chromosome 7; and 2q22-q24 (for *GRB14*), in the middle part of the long arm of chromosome 2, are shown. See text for additional details.

## ABBREVIATIONS

Akt: = v-*akt* murine thymoma viral oncogene homolog
BPS: = Between PH and SH2
*COBL*: = Cordon-bleu gene
*DDC*: = DOPA-decarboxylase gene
EGFR: = Epidermal growth factor receptor
eIF4E: = Eukaryotic initiation factor 4E
Eph: = Erythropoietin-producing hepatocellular carcinoma cells
ER: = Estrogen receptor
*ERBB*: = Erythroblastic leukemia viral oncogene homolog
FAK: = Focal adhesion kinase
Grb: = Growth factor receptor bound
IGF-I: = Insulin-like growth factor-I
IR: = Insulin receptor
*KIT*: = v-*kit* Hardy-Zuckerman 4 feline sarcoma viral oncogene homolog
matUPD: = Maternal UPD
matUPD7: = matUPD of chromosome 7
PDGF-BB: = Platelet-derived growth factor-BB
patUPD: = Paternal UPD
PH: = Pleckstrin homology
PI3K: = Phosphatidylinositol 3-kinase
PR: = Proline-rich
RA: = Ras-associating
SH2: = Src homology 2
SH3: = Src homology 3
SNP: = Single-nucleotide polymorphism
UPD: = Uniparental disomy
5’-UTR: = 5’-untraslated region
